# GammaTile for Gliomas: A Single-Center Case Series

**DOI:** 10.7759/cureus.19390

**Published:** 2021-11-09

**Authors:** Hailey C Budnick, Angela M Richardson, Kevin Shiue, Gordon Watson, Sook K Ng, Yi Le, Mitesh V Shah

**Affiliations:** 1 Neurological Surgery, Indiana University Health, Indianapolis, USA; 2 Radiation Oncology, Indiana University (IU) Health Simon Cancer Center, Indianapolis, USA

**Keywords:** cesium 131, surgically implanted radiotherapy, recurrent, glioblastoma, gliomas, brachytherapy, gamma tile

## Abstract

GammaTile® (GT Medical Technologies, Tempe, Arizona) is a surgically targeted radiation source, approved by FDA for brachytherapy in primary and secondary brain neoplasms. Each GammaTile is composed of a collagen sponge with four seeds of cesium 131 and is particularly useful in recurrent tumors. We report our early experience in seven patients with recurrent gliomas to assess this type of brachytherapy with particular attention to ease of use, complication, and surgical planning.

This study represents a retrospective chart review of surgical use and early clinical outcomes of GammaTile in recurrent gliomas. The number of tiles was planned using pre-operative imaging and dosimetry was planned based on post-operative imaging. Patients were followed during their hospital stay and were followed up after discharge. Parameters such as case length, resection extent, complication, ICU length of stay (LOS), hospital LOS, pre-operative Glasgow Coma Scale (GCS), immediate post-operative GCS, post-operative imaging findings, recurrence at follow-up, length of follow-up, and dosimetry were collected in a retrospective manner.

Seven patients were identified that met the inclusion criteria. Two patients were diagnosed with recurrent glioblastoma multiforme (GBM), one lower-grade glioma that recurred as a GBM, one GBM that recurred as a gliosarcoma, and two recurrent oligodendrogliomas. We found that operation time, ICU LOS, hospital LOS, pre- and post-operative GCS, and post-operative complications were within the expected ranges for tumor resection patients. Further, dosimetry data suggests that six out of seven patients received adequate radiation coverage, with the seventh having implantation limitations due to nearby organs at risk. We report no postoperative complications that can be attributed to the GammaTiles themselves.

In our cohort, we report seven cases where GammaTiles were implanted in recurrent gliomas. No implant-related post-operative complications were identified. This early data suggests that GammaTile can be a safe form of brachytherapy in recurrent gliomas.

## Introduction

Gliomas are among the most common primary brain tumors and are the most common malignant primary brain tumor [1]. There are many subtypes of glioma, now categorized by a combination of histopathologic findings and molecular genetics [2]. These subtypes are divided into four grades with grade three (i.e. anaplastic astrocytoma, anaplastic oligodendroglioma) and four (glioblastoma) considered high-grade tumors. Despite cytoreduction followed by chemotherapy and radiation, life expectancy is limited and recurrence is quite common [3]. The neurosurgical and medical communities have studied how best to care for these patients in order to extend their lives and maximize their quality of life. The most consistent prognostic factors in glioma resection are age, extent of resection, and Karnofsky performance status [4,5].

Numerous clinical trials have been performed to help prolong survival, many with marginal success. The Stupp protocol has been adopted via level I evidence of increased survival as the primary post-surgical treatment of glioblastoma multiforme (GBM). This protocol includes external beam radiotherapy in the form of fractionated focal irradiation in daily fractions of 2Gy given five days per week for six weeks, for a total of 60Gy. The radiation is accompanied by concurrent and post-radiation temozolomide [6]. The post-surgical adjuvant treatment regimen for high-grade gliomas, especially recurrent tumors, has remained controversial. The most important treatment for high-grade gliomas is fractionated radiotherapy, typically at a dose of 54Gy to 60Gy [7]. It has been observed that survival is shorter in elderly patients with glioblastoma, on average seven to nine months [8,9]. It has been widely accepted that elderly patients have higher complication rates from higher radiation doses.

The role of radiation in the treatment of high-grade gliomas has been well-established. Following surgery, radiotherapy is typically delayed several weeks in order to allow for wound healing. Given the aggressive nature of recurrent tumors, this delay may allow for interval disease progression [10]. There is no consensus as to the appropriate modality of radiation in recurrent gliomas. On the one hand, external beam radiation can cover a large surface area making it attractive for invasive high-grade lesions in which the tumor cells can be expected to have infiltrated beyond the contrast-enhancing portion on MRI. However, the morbidity associated with external beam radiation, and significantly more so whole brain radiation, can be significant. Stereotactic radiosurgery (SRS) is an attractive alternative that allows for focused radiotherapy to a small, targeted area. This is ideal for small lower-grade lesions and small recurrent or residual tumors following resection. However, it is thought to be inadequate for complete cellular control in invasive histology, such as that found in high-grade tumors [11]. SRS may be used in recurrent GBM since those patients routinely receive fractionated radiotherapy as part of the Stupp protocol; due to its focused nature, SRS may mitigate the toxicity and subsequent radiation necrosis that can be seen with repeat fractionated radiotherapy to a wider field. In many patients, initial radiotherapy delivers large enough doses that treatment with further radiation is precluded in order to prevent radiation brain injury [12]. Because of these limitations, repeat radiotherapy in recurrent gliomas is typically reserved for patients with tumors in non-eloquent areas, aggressive lesions, or in palliative cases in whom life expectancy is presumed to be very short [13]. When recurrent radiotherapy is pursued, in order to ensure that all parenchymal invasion is included, a wider margin is usually required which increases the risk to surrounding tissue [14].

Brachytherapy has been introduced as an alternative to external beam radiotherapy. One attractive feature of brachytherapy is that it is performed at the time of tumor resection, with no delay in radiation delivery to the tumor bed. Brachytherapy may be given in the form of yttrium, iodine, iridium, or cesium isotopes contained in an implantable matrix allowing for focal irradiation. This allows for both surgical resection and radiotherapy to be completed in one session, which has multiple advantages including immediate radiotherapy to these patients, logistical convenience without repeated trips to a center for radiation treatment, less radiation damage to surrounding tissue, and the ability to use higher targeted doses of radiation along the tumor bed [15]. Limiting radiation to surrounding tissues may be advantageous in reducing rates of radiation necrosis and in protecting the skin overlying the surgical site from radiation-induced damage. Placing radioisotopes encased in seeds along the areas of concern under direct visualization may be more accurate than immediate post-operative imaging in determining the areas most in need of additional treatments [16,17]. The use of brachytherapy has been established as part of the care regimen at many institutions and has been shown to be a safe and effective tool both in initial and re-operations [15,18,19,20,21]. While the success of multiple trials of brachytherapy with radioisotope seeds has been described, one limitation has been the occurrence of radiation necrosis associated with inconsistent radiation therapy to the tumor bed. Since the seeds are placed directly onto the brain surface, the area of the brain in direct contact with the seeds may receive supratherapeutic doses and the remaining brain may receive subtherapeutic doses [22].

To alleviate these difficulties, GammaTile® (GT Medical Technologies, Tempe, Arizona) is a permanently implanted collagen sponge with four Cesium-131 titanium seeds. These tiles are placed along the resection bed of malignant tumors. The seeds are embedded within these sponges with specific geometry. This allows for precise spacing of the radiation sources with predictable dosimetry and provides a stable substrate so that the sources do not shift following implantation. The collagen matrix provides a barrier to prevent unintended radiation injury from direct contact with the brain. These tiles are permanently implanted and have a short half-life (9.7 days) and with a dose of 80-120Gy in the first few millimeters of the contacted brain but without the supratherapeutic dose of over 2000Gy that has been seen in traditional brachytherapy seeds [22]. This allows for a therapeutic dose of radiation to be delivered to the area of concern within minutes of maximal safe resection without the harmful effects of supratherapeutic dosing including radiation necrosis as well as minimizing sub-optimal treatments to areas not in contact with the seeds. GammaTile is FDA approved first for recurrent intracranial neoplasms and recently expanded to use during the initial tumor resection.

## Case presentation

Methods

Patient Selection And Data Collection

Following IRB approval, all patients who underwent craniotomy for glioma resection with GammaTile placement at the time of surgery at our institution were identified. Demographic, clinical, and tumor data including age, sex, pre-operative Glasgow Coma Scale (GCS), post-operative GCS, length of surgery (LOS), ICU LOS, hospital LOS, post-operative disposition, length of follow-up, and presence of post-operative recurrence or radiation necrosis were extracted from the medical record. The presence of recurrence and need for re-resection was determined by multidisciplinary tumor board composed of neurosurgery, neuro-oncology, radiology, and radiation-oncology.

Pre-operative Planning

All patients underwent the standard pre-operative imaging and diagnostic workup and underwent maximal safe resection by a single surgeon at Indiana University. Pre-operative planning with the surgeon, radiation oncologists, and physicists was performed to decide how many GammaTiles to lay along the resection bed. The tumor cavity was expected to shrink, and this was taken into account in the planning process.

Surgical Treatment

All patients had the tiles placed by the primary surgeon using the appropriate protective equipment. The patients received the usual post-operative care with brief post-operative stay in the ICU and post-operative CT and MRI.

Post-operative Imaging and Dosimetry

All patients received post-operative imaging to assess for extent of resection and to assess for any post-operative hematomas or new mass lesions. Post-operative MRI and CT images were imported into the brachytherapy planning software (MIM Software Inc. Cleveland, OH 44122) and co-registered to each other. Radioactive seeds were identified by medical physicists in CT images and radiation doses were calculated per the American Association of Physicists in Medicine (AAPM) TG-43 protocol. The resection cavity was outlined using seeds as a surrogate. The residual tumor, identified as contrast enhanced areas in the post-operative T1 + C MRI images, was contoured by radiation oncologists and defined as gross target volume of residual tumor (GTVr). The planning target volume (PTV) is defined as the union of GTVr and 5mm expansion of cavity volume. The goal of brachytherapy is to deliver 60Gy radiation to the PTV. The volume of both GTVr and PTV and their corresponding dosimetry parameters were calculated and analyzed. The radiation doses to organs at risk (OAR) such as the brainstem, optical nerves, and chiasm were also calculated.

Results

Seven patients meeting inclusion criteria were identified; four were males and three were females. Their ages ranged from 33-68 years with a mean age of 54 years old (Table 1). Three were initially resected GBMs, two were anaplastic astrocytomas, one of which had progressed to GBM after the initial operation and one of which had not. Interestingly, one of the initial GBM resections had gliosarcoma pathology on the second resection. One WHO grade II oligodendroglioma that had progressed to anaplastic after the first resection and one anaplastic astrocytoma that was a WHO grade II oligodendroglioma on the second resection. All patients but the WHO grade II oligodendroglioma had external beam radiation prior to their initial resection and all but the two oligodendrogliomas had been on temozolomide. Other common adjuvant therapies of the first operation included bevacizumab (Avastin®), pembrolizumab (Keytruda®), and procarbazine-lomustine-vincristine (PVC).

**Table 1:** Patient demographics and data from initial surgery GBM: glioblastoma multiforme; mo: month; N/A: not applicable

Patient	1	2	3	4	5	6	7
Age	53	36	65	33	68	68	53
Sex	Male	Female	Female	Male	Male	Male	Female
Initial pathology	GBM (WHO grade 4)	GBM (WHO grade 4)	Anaplastic astrocytoma (WHO grade 3)	Anaplastic astrocytoma (WHO grade 3)	Oligodendroglioma (WHO grade 2)	GBM (WHO grade 4)	Anaplastic oligodendroglioma (WHO grade 3)
Complications from initial surgery	N/A	Significant radiation necrosis	Radiation necrosis, residual tumor, second resection	N/A	N/A	N/A	N/A
Time to follow-up	8 mo	3 mo	2 mo	1 mo	2 mo	1 mo	Upcoming post-op appointment

All re-resections and GammaTile placements were in response to symptomatic recurrence of their primary tumors, and all were performed between February to October 2021 by a single surgeon at a single academic center. Between five to nine GammaTiles were placed in the resection cavity and this number was determined by pre-operative imaging and intra-operative findings and was the combined decision of the neurosurgical staff and radiation oncology staff who was available intra-operatively. The length of surgery ranged from 150 to 268 minutes with a mean of 225 minutes. Gross total resection (GTR) was achieved in four patients and subtotal resection was achieved in the other three.

GammaTile brachytherapy was not associated with increased rate of complications. No patients developed infections, significant pseudomeningocele, cerebrospinal fluid leak, thromboembolic events, or readmission within 30 days. Post-operatively, one patient’s course was complicated by significant post-operative cerebral edema requiring hypertonic saline, increased steroid therapy, and an extended ICU stay. The cerebral edema was thought to be due to her significant pre-operative cerebral edema as well as the subtotal resection. One other patient had significant post-operative expressive aphasia, and another had significant post-operative weakness but these results were expected given the location of the tumors. ICU LOS ranged from one to four days and hospital LOS ranged from two to eleven days. Four patients went home after discharge and three patients went to an acute rehabilitation facility (Table 2).

**Table 2:** Data at recurrence Tx: Treatment; STR: Subtotal resection; GTR: Gross total resection; GCS: Glasgow Coma Scale; LOS: Length of stay; L: Left; NaCl: sodium chloride

Patient	1	2	3	4	5	6	7
Second tx adjuvants	Carboplatin Bevacizumab	Pembrolizumab Bevacizumab	None	Lomustin	None	None	None
Extent of resection	GTR	STR	GTR	STR	STR	GTR	GTR
GCS Preop/Postop	15/15	15/14	15/13	15/14	15/14	15/15	15/14
ICU/ hospital LOS	1/2	2/2	4/8	1/7	1/11	1/3	2/4
Disposition	Home	Home	Rehab	Rehab	Rehab	Home	Home
Subsequent recurrence	Yes	Yes	Maybe	Yes	No	Maybe	Unknown
Complications	None	None	Significant postop edema, requiring additional Decadron® and 3% NaCl	Expressive aphasia	L hemiparesis	None	None
Radiation necrosis	None	None	None	None	None	None	Unknown

On follow-up imaging, three patients had evidence of tumor progression or recurrence and two had MRI findings concerning progression or recurrence versus enhancement of post-operative debris or the GammaTiles themselves. Of the patients with presumed recurrence on follow-up imaging, one of them had a GTR and the other two received subtotal resections. One patient had a GTR without recurrence on repeat imaging. One patient has not yet had their follow-up imaging. No evidence of radiation necrosis was seen on any post-operative imaging.

Following the intra-operative placement of the GammaTiles, the radiation oncological team calculated dosimetry parameters (Table 3). The average of GTVr and PTV volume was 11.5cc and 48.4cc, respectively. D90 (dose covers 90% target volume) and V100 (target volume covered by 100% prescription dose) of GTVr and PTV are shown in Table 3. The average of D90 of GTVr and PTV are 117% and 97%, respectively. The average of V100 of GTVr and PTV are 88% and 89%, respectively. Three out of seven cases have no organs at risk (OARs) near the resection cavities. Brainstem doses are less than 60Gy for one case and less than 15Gy for two cases. One case with a left frontal tumor has optical nerve and chiasm doses less than 30 Gy.

**Table 3:** GammaTile data GT: GammaTile; GTVr: Gross total volume of the residual tumor; PTV: Planning target volume; L: Left; R: Right; Min: Minutes; Vol: volume

Patient	1	2	3	4	5	6	7
Number of GT (seeds)	5 (20)	7 (28)	7 (28)	9 (36)	8 (32)	6 (24)	4 (16)
Vol_GTVr (cc)	17.7	26.2	5.1	13	0.1	18.1	No residual
D90_GTVr (%)	148	134	90.7	30	135	163	No residual
V100_GTVr (%)	100	100	84.4	29	100	100	No residual
Vol_PTV (cc)	35.6	53.6	55.9	78.8	60	29.1	25.9
D90_PTV (%)	99.5	88	96.3	70	102.3	131	96
V100_PTV (%)	90	83	87.9	81.3	90.7	99.1	87.9
Organs at risk nearby	None	None	None, Brainstem max <15Gy (25% Rx)	Brainstem max <60Gy	None, Brainstem max <15Gy (25% Rx)	None	Optical nerve, chiasm max <30Gy (50% Rx)
			Location		L frontal			L frontotemporal	L parietooccipital	L parietotemporal	R parietooccipital	R frontoparietal	L frontal
Surgery length (min)	248	244	222	267	177	150	268

## Discussion

Surgically implanted brachytherapy has shown promise for multiple tumor types over the past several years. A single-arm, single-center prospective clinical trial was performed on 20 high-grade recurrent meningiomas. This study found statistically significant improvement in time-to-local disease progression compared to the same patient following their initial operation. At 18 months, 89% of patients had no disease progression and the disease progression was found to be 11 months longer in those that did progress [14]. With these results, a two-center (Barrow Neurologic Institute and St. Joseph’s Hospital in Phoenix, AZ) larger trial was performed including gliomas, metastatic lesions, and other primary neoplasms [23]. The result was a similar disease control with complication rates that were similar or improved compared to the literature for surgical and radiation adverse events [24]. Specifically, the rate of radiation necrosis or radiation-related brain injury was found to be 7.6%, which is lower than many reported studies with external beam or traditional brachytherapy studies [25,26,27].

In our case series, we describe the placement and early outcomes of seven patients with recurrent gliomas in which GammaTile brachytherapy was implanted. We reported no post-operative complications due to the implantation itself. This single-center series demonstrates promising early results of surgically implanted GammaTile brachytherapy. GammaTiles were able to be implanted in all cases for which they were planned. The complication rates were within the expected range. Four patients had a completely smooth post-operative course. One patient with the tumor abutting the motor strip had post-operative left hemiparesis, which improved with time. Another patient had moderate expressive aphasia post-operatively, but this was expected given his tumor abutting Broca’s area. Another patient had significant post-operative edema requiring additional steroids and hypertonic saline but did not require any additional procedures and the patient had no lasting neurological deficits. None of these complications were thought to be due to the GammaTile placement.

In our seven cases, we found GammaTile brachytherapy to provide adequate radiation dose coverage to the resection cavities, demonstrated by the fact that all PTV V100 are above 80%. The residual tumors were well covered in six out of seven patients in this cohort. The only case in which the residual tumor was not well covered was one in which the disease had progressed much further beyond the pre-operative planned resection area. The intra-operative decision was made not to resect those progressed regions due to their locations. There is no extra high dose area beyond resection cavities and all OARs doses are within tolerance.

Unlike in previous brachytherapy and GammaTile trials, none of our patients have shown signs of radiation necrosis on surveillance imaging. The GammaTile portion of the case was very streamlined and convenient without any of the reported difficulty with handling the brachytherapy seeds as described in prior trials [28] and did not significantly lengthen the overall case length. We reported no deaths in our cohort and all patients were discharged to either home or a rehabilitation center within 11 days of surgery. However, our data is preliminary and further follow-up will be required to determine true rates of radiation-induced brain injury. In fact, one of the patients had not even had their first post-operative clinic visit at the time of publication of this study. In addition to that, our sample size is too small to make any generalizable claims. Despite these limitations, we find that our data support that the implantation of GammaTile can be a safe adjuvant for recurrent gliomas.

## Conclusions

We present preliminary data of our experience with GammaTile brachytherapy in recurrent gliomas. We provide dosimetry data supporting that this form of brachytherapy provides adequate radiation coverage to the resection bed while not giving supratherapeutic doses to contacting brain and without harming nearby OAR. We reported no complications associated with the placement of the GammaTiles. We also showed no radiation necrosis on our early follow-up imaging. The placement of the GammaTiles added very little time to the case length and was a very convenient and streamlined process. The patients who received GammaTile placement had expected post-operative courses similar to our experience with tumor resection patients including ICU and hospital LOS, post-operative complications, and discharge disposition. This early data suggests that GammaTile is a safe form of brachytherapy in recurrent gliomas.
